# Online distance learning in hand therapy

**DOI:** 10.1186/1753-6561-9-S3-A111

**Published:** 2015-05-19

**Authors:** Nathan Vytialingam

**Affiliations:** 1 School of Occupational Therapy, Perdana University, Serdang, 43400, Malaysia

## Introduction

Hand Therapy is a sub speciality area within the field of orthopaedics rehabilitation that encompasses hand therapists that may have a background as occupational therapist or physiotherapist. It requires advanced continuing graduate education, clinical experience and independent study. Available hand therapy programs are time-consuming, expensive and inaccessible. The authors conceptualised and developed a modular and flexible online and distance learning program for knowledge acquisition that is university accessible and affordable.

## Methodology

The program was developed by using an Instructional Design model (ADDIE). The Delphi method was used to map the curriculum grounded on community orientated and outcome based, incorporating, rehabilitation and surgical experts using data from the tertiary hand referral centre. The pedagogy chosen catered to the projected learner demographics utilising the work based learning framework ensuring flexibility, focusing on process-driven curriculum, self-directed and experiential learning. Assignments, automated e-assessments, learning logs and reflective practice were identified for formative assessment.

## Results

An on-line modular programme was drafted offering three levels of qualifications: PG Certificate, Diploma and Master’s degree, allowing flexibility and self-paced learning. The MOODLE open source learning management system was chosen to deliver the program. An International faculty was recruited using the authors network and the program was deployed on line for evaluation (handtherapyedu.com). Perdana University was partnered for program accreditation.

## Summary

The authors have shown that it is possible for communities of practice to develop and deploy universally accessible and affordable work based learning programs. Everyone can learn. The scope of growth with ubiquitous learning will change the landscape for graduate education. We believe massively open online course (MOOC), delivering learning content online to virtually anyone, will be the model for the future and will alter the relationship between learner, teacher, university and all stake holders of education. There are early evidence of the viability of this educational model.

